# Supplementary material for: Overlapping cell population expression profiling and regulatory inference in C. elegans
Source: BMC Genomics. 2016 Feb 29;17:159. doi: 10.1186/s12864-016-2482-z (PMC4772325; doi:10.1186/s12864-016-2482-z)
Supplement: Additional file 13: — Web supplement. (DOC 21 kb) [file 12864_2016_2482_MOESM13_ESM.zip › sortWeb/clusters/hier.300.clusters/72.html]

Cluster 72 

## Cluster 72

### Expression

| cnd-1 rep. 1 | cnd-1 rep. 2 | cnd-1 rep. 3 | pha-4 rep. 1 | pha-4 rep. 2 | pha-4 rep. 3 | ceh-27 | ceh-36 | ceh-6 | F21D5.9 | mir-57 | mls-2 | pal-1 | pros-1 | ttx-3 | unc-130 | hlh-16 | irx-1 | ceh-6 (+) hlh-16 (+) | ceh-6 (+) hlh-16 (-) | ceh-6 (-) hlh-16 (+) | cnd-1 singlets | pha-4 singlets | 0 | 60 | 120 | 150 | 180 | 240 | 330 | 390 | 420 | 480 | 540 | 570 | 600 | 630 | 660 | NAME | Functional description |
| --- | --- | --- | --- | --- | --- | --- | --- | --- | --- | --- | --- | --- | --- | --- | --- | --- | --- | --- | --- | --- | --- | --- | --- | --- | --- | --- | --- | --- | --- | --- | --- | --- | --- | --- | --- | --- | --- | --- | --- |
|  |  |  |  |  |  |  |  |  |  |  |  |  |  |  |  |  |  |  |  |  |  |  |  |  |  |  |  |  |  |  |  |  |  |  |  |  |  | *mnp-1* | Matrix Non-Peptidase homolog |
|  |  |  |  |  |  |  |  |  |  |  |  |  |  |  |  |  |  |  |  |  |  |  |  |  |  |  |  |  |  |  |  |  |  |  |  |  |  | *epi-1* | abnormal EPIthelia |
|  |  |  |  |  |  |  |  |  |  |  |  |  |  |  |  |  |  |  |  |  |  |  |  |  |  |  |  |  |  |  |  |  |  |  |  |  |  | *fbxc-24* | F-box C protein |
|  |  |  |  |  |  |  |  |  |  |  |  |  |  |  |  |  |  |  |  |  |  |  |  |  |  |  |  |  |  |  |  |  |  |  |  |  |  | *cbs-1* | Cystathionine Beta-Synthase |
|  |  |  |  |  |  |  |  |  |  |  |  |  |  |  |  |  |  |  |  |  |  |  |  |  |  |  |  |  |  |  |  |  |  |  |  |  |  | *pcp-5* | Prolyl Carboxy Peptidase like |
|  |  |  |  |  |  |  |  |  |  |  |  |  |  |  |  |  |  |  |  |  |  |  |  |  |  |  |  |  |  |  |  |  |  |  |  |  |  | D2045.9 |  |
|  |  |  |  |  |  |  |  |  |  |  |  |  |  |  |  |  |  |  |  |  |  |  |  |  |  |  |  |  |  |  |  |  |  |  |  |  |  | *efhd-1* | EF HanD calcium binding protein |
|  |  |  |  |  |  |  |  |  |  |  |  |  |  |  |  |  |  |  |  |  |  |  |  |  |  |  |  |  |  |  |  |  |  |  |  |  |  | *calu-1* | CALUmenin (calcium-binding protein) homolog |
|  |  |  |  |  |  |  |  |  |  |  |  |  |  |  |  |  |  |  |  |  |  |  |  |  |  |  |  |  |  |  |  |  |  |  |  |  |  | Y46G5A.22 |  |
|  |  |  |  |  |  |  |  |  |  |  |  |  |  |  |  |  |  |  |  |  |  |  |  |  |  |  |  |  |  |  |  |  |  |  |  |  |  | *hnd-1* | HAND bHLH transcription factor family |
|  |  |  |  |  |  |  |  |  |  |  |  |  |  |  |  |  |  |  |  |  |  |  |  |  |  |  |  |  |  |  |  |  |  |  |  |  |  | Y65B4BL.7 |  |
|  |  |  |  |  |  |  |  |  |  |  |  |  |  |  |  |  |  |  |  |  |  |  |  |  |  |  |  |  |  |  |  |  |  |  |  |  |  | *arrd-14* | ARRestin Domain protein |
|  |  |  |  |  |  |  |  |  |  |  |  |  |  |  |  |  |  |  |  |  |  |  |  |  |  |  |  |  |  |  |  |  |  |  |  |  |  | *pyc-1* | PYruvate Carboxylase |
|  |  |  |  |  |  |  |  |  |  |  |  |  |  |  |  |  |  |  |  |  |  |  |  |  |  |  |  |  |  |  |  |  |  |  |  |  |  | *pck-2* | Phosphoenolypyruvate CarboxyKinase |
|  |  |  |  |  |  |  |  |  |  |  |  |  |  |  |  |  |  |  |  |  |  |  |  |  |  |  |  |  |  |  |  |  |  |  |  |  |  | *sqrd-1* | Sulfide Quinone oxidoReDuctase |
|  |  |  |  |  |  |  |  |  |  |  |  |  |  |  |  |  |  |  |  |  |  |  |  |  |  |  |  |  |  |  |  |  |  |  |  |  |  | *cpl-1* | CathePsin L family |
|  |  |  |  |  |  |  |  |  |  |  |  |  |  |  |  |  |  |  |  |  |  |  |  |  |  |  |  |  |  |  |  |  |  |  |  |  |  | Y53G8B.2 |  |
|  |  |  |  |  |  |  |  |  |  |  |  |  |  |  |  |  |  |  |  |  |  |  |  |  |  |  |  |  |  |  |  |  |  |  |  |  |  | *mdh-1* | Malate DeHydrogenase |
|  |  |  |  |  |  |  |  |  |  |  |  |  |  |  |  |  |  |  |  |  |  |  |  |  |  |  |  |  |  |  |  |  |  |  |  |  |  | C44B7.10 |  |
|  |  |  |  |  |  |  |  |  |  |  |  |  |  |  |  |  |  |  |  |  |  |  |  |  |  |  |  |  |  |  |  |  |  |  |  |  |  | *gyg-1* | GlYcoGenin like |
|  |  |  |  |  |  |  |  |  |  |  |  |  |  |  |  |  |  |  |  |  |  |  |  |  |  |  |  |  |  |  |  |  |  |  |  |  |  | T05F1.8 |  |
|  |  |  |  |  |  |  |  |  |  |  |  |  |  |  |  |  |  |  |  |  |  |  |  |  |  |  |  |  |  |  |  |  |  |  |  |  |  | *ceh-49* | C. Elegans Homeobox |
|  |  |  |  |  |  |  |  |  |  |  |  |  |  |  |  |  |  |  |  |  |  |  |  |  |  |  |  |  |  |  |  |  |  |  |  |  |  | C43C3.4 |  |
|  |  |  |  |  |  |  |  |  |  |  |  |  |  |  |  |  |  |  |  |  |  |  |  |  |  |  |  |  |  |  |  |  |  |  |  |  |  | *cri-2* | Conserved Regulator of Innate immunity |
|  |  |  |  |  |  |  |  |  |  |  |  |  |  |  |  |  |  |  |  |  |  |  |  |  |  |  |  |  |  |  |  |  |  |  |  |  |  | F13D12.10 |  |
|  |  |  |  |  |  |  |  |  |  |  |  |  |  |  |  |  |  |  |  |  |  |  |  |  |  |  |  |  |  |  |  |  |  |  |  |  |  | Y55F3BR.6 |  |
|  |  |  |  |  |  |  |  |  |  |  |  |  |  |  |  |  |  |  |  |  |  |  |  |  |  |  |  |  |  |  |  |  |  |  |  |  |  | *bro-1* | BROther (Drosophila tx factor partner) homolog |
|  |  |  |  |  |  |  |  |  |  |  |  |  |  |  |  |  |  |  |  |  |  |  |  |  |  |  |  |  |  |  |  |  |  |  |  |  |  | ZC247.1 |  |
|  |  |  |  |  |  |  |  |  |  |  |  |  |  |  |  |  |  |  |  |  |  |  |  |  |  |  |  |  |  |  |  |  |  |  |  |  |  | T23B12.11 |  |
|  |  |  |  |  |  |  |  |  |  |  |  |  |  |  |  |  |  |  |  |  |  |  |  |  |  |  |  |  |  |  |  |  |  |  |  |  |  | *clec-149* | C-type LECtin |
|  |  |  |  |  |  |  |  |  |  |  |  |  |  |  |  |  |  |  |  |  |  |  |  |  |  |  |  |  |  |  |  |  |  |  |  |  |  | *best-6* | BESTrophin (chloride channel) homolog |
|  |  |  |  |  |  |  |  |  |  |  |  |  |  |  |  |  |  |  |  |  |  |  |  |  |  |  |  |  |  |  |  |  |  |  |  |  |  | *linc-43* | Long Intervening Non-Coding RNA |
|  |  |  |  |  |  |  |  |  |  |  |  |  |  |  |  |  |  |  |  |  |  |  |  |  |  |  |  |  |  |  |  |  |  |  |  |  |  | T02G5.4 |  |
|  |  |  |  |  |  |  |  |  |  |  |  |  |  |  |  |  |  |  |  |  |  |  |  |  |  |  |  |  |  |  |  |  |  |  |  |  |  | *gpx-6* | Glutathione PeroXidase |
|  |  |  |  |  |  |  |  |  |  |  |  |  |  |  |  |  |  |  |  |  |  |  |  |  |  |  |  |  |  |  |  |  |  |  |  |  |  | *rnt-1* | RuNT related |
|  |  |  |  |  |  |  |  |  |  |  |  |  |  |  |  |  |  |  |  |  |  |  |  |  |  |  |  |  |  |  |  |  |  |  |  |  |  | K05F1.5 |  |
|  |  |  |  |  |  |  |  |  |  |  |  |  |  |  |  |  |  |  |  |  |  |  |  |  |  |  |  |  |  |  |  |  |  |  |  |  |  | *linc-17* | Long Intervening Non-Coding RNA |
|  |  |  |  |  |  |  |  |  |  |  |  |  |  |  |  |  |  |  |  |  |  |  |  |  |  |  |  |  |  |  |  |  |  |  |  |  |  | *zmp-1* | Zinc MetalloProtease |
|  |  |  |  |  |  |  |  |  |  |  |  |  |  |  |  |  |  |  |  |  |  |  |  |  |  |  |  |  |  |  |  |  |  |  |  |  |  | C04C11.1 |  |
|  |  |  |  |  |  |  |  |  |  |  |  |  |  |  |  |  |  |  |  |  |  |  |  |  |  |  |  |  |  |  |  |  |  |  |  |  |  | *gei-15* | GEX Interacting protein |
|  |  |  |  |  |  |  |  |  |  |  |  |  |  |  |  |  |  |  |  |  |  |  |  |  |  |  |  |  |  |  |  |  |  |  |  |  |  | *tni-1* | TropoNin I |
|  |  |  |  |  |  |  |  |  |  |  |  |  |  |  |  |  |  |  |  |  |  |  |  |  |  |  |  |  |  |  |  |  |  |  |  |  |  | D2092.4 |  |
|  |  |  |  |  |  |  |  |  |  |  |  |  |  |  |  |  |  |  |  |  |  |  |  |  |  |  |  |  |  |  |  |  |  |  |  |  |  | F44B9.2 |  |
|  |  |  |  |  |  |  |  |  |  |  |  |  |  |  |  |  |  |  |  |  |  |  |  |  |  |  |  |  |  |  |  |  |  |  |  |  |  | K02A11.3 |  |
|  |  |  |  |  |  |  |  |  |  |  |  |  |  |  |  |  |  |  |  |  |  |  |  |  |  |  |  |  |  |  |  |  |  |  |  |  |  | *clec-233* | C-type LECtin |
|  |  |  |  |  |  |  |  |  |  |  |  |  |  |  |  |  |  |  |  |  |  |  |  |  |  |  |  |  |  |  |  |  |  |  |  |  |  | *cyp-23A1* | CYtochrome P450 family |
|  |  |  |  |  |  |  |  |  |  |  |  |  |  |  |  |  |  |  |  |  |  |  |  |  |  |  |  |  |  |  |  |  |  |  |  |  |  | *pho-14* | intestinal acid PHOsphatase |
|  |  |  |  |  |  |  |  |  |  |  |  |  |  |  |  |  |  |  |  |  |  |  |  |  |  |  |  |  |  |  |  |  |  |  |  |  |  | *pqn-48* | Prion-like-(Q/N-rich)-domain-bearing protein |
|  |  |  |  |  |  |  |  |  |  |  |  |  |  |  |  |  |  |  |  |  |  |  |  |  |  |  |  |  |  |  |  |  |  |  |  |  |  | Y51A2D.14 |  |
|  |  |  |  |  |  |  |  |  |  |  |  |  |  |  |  |  |  |  |  |  |  |  |  |  |  |  |  |  |  |  |  |  |  |  |  |  |  | *mlc-2* | Myosin Light Chain |
|  |  |  |  |  |  |  |  |  |  |  |  |  |  |  |  |  |  |  |  |  |  |  |  |  |  |  |  |  |  |  |  |  |  |  |  |  |  | *pat-10* | Paralysed Arrest at Two-fold |
|  |  |  |  |  |  |  |  |  |  |  |  |  |  |  |  |  |  |  |  |  |  |  |  |  |  |  |  |  |  |  |  |  |  |  |  |  |  | C54E4.2 |  |
|  |  |  |  |  |  |  |  |  |  |  |  |  |  |  |  |  |  |  |  |  |  |  |  |  |  |  |  |  |  |  |  |  |  |  |  |  |  | *cpn-3* | CalPoNin |
|  |  |  |  |  |  |  |  |  |  |  |  |  |  |  |  |  |  |  |  |  |  |  |  |  |  |  |  |  |  |  |  |  |  |  |  |  |  | *mlc-3* | Myosin Light Chain |
|  |  |  |  |  |  |  |  |  |  |  |  |  |  |  |  |  |  |  |  |  |  |  |  |  |  |  |  |  |  |  |  |  |  |  |  |  |  | *pfn-3* | ProFiliN |
|  |  |  |  |  |  |  |  |  |  |  |  |  |  |  |  |  |  |  |  |  |  |  |  |  |  |  |  |  |  |  |  |  |  |  |  |  |  | *sgcb-1* | SarcoGlyCan Beta homolog |
|  |  |  |  |  |  |  |  |  |  |  |  |  |  |  |  |  |  |  |  |  |  |  |  |  |  |  |  |  |  |  |  |  |  |  |  |  |  | *sgca-1* | SarcoGlyCan Alpha homolog |
|  |  |  |  |  |  |  |  |  |  |  |  |  |  |  |  |  |  |  |  |  |  |  |  |  |  |  |  |  |  |  |  |  |  |  |  |  |  | *sgn-1* | SarcoGlycaN |
|  |  |  |  |  |  |  |  |  |  |  |  |  |  |  |  |  |  |  |  |  |  |  |  |  |  |  |  |  |  |  |  |  |  |  |  |  |  | *dim-1* | DIsorganized Muscle |
|  |  |  |  |  |  |  |  |  |  |  |  |  |  |  |  |  |  |  |  |  |  |  |  |  |  |  |  |  |  |  |  |  |  |  |  |  |  | C29F5.1 |  |
|  |  |  |  |  |  |  |  |  |  |  |  |  |  |  |  |  |  |  |  |  |  |  |  |  |  |  |  |  |  |  |  |  |  |  |  |  |  | H12D21.9 |  |
|  |  |  |  |  |  |  |  |  |  |  |  |  |  |  |  |  |  |  |  |  |  |  |  |  |  |  |  |  |  |  |  |  |  |  |  |  |  | F36F2.1 |  |
|  |  |  |  |  |  |  |  |  |  |  |  |  |  |  |  |  |  |  |  |  |  |  |  |  |  |  |  |  |  |  |  |  |  |  |  |  |  | *sfxn-5* | SideroFleXiN (mitochondrial iron transporter) |
|  |  |  |  |  |  |  |  |  |  |  |  |  |  |  |  |  |  |  |  |  |  |  |  |  |  |  |  |  |  |  |  |  |  |  |  |  |  | F40A3.6 |  |
|  |  |  |  |  |  |  |  |  |  |  |  |  |  |  |  |  |  |  |  |  |  |  |  |  |  |  |  |  |  |  |  |  |  |  |  |  |  | B0310.1 |  |
|  |  |  |  |  |  |  |  |  |  |  |  |  |  |  |  |  |  |  |  |  |  |  |  |  |  |  |  |  |  |  |  |  |  |  |  |  |  | *mig-6* | abnormal cell MIGration |
|  |  |  |  |  |  |  |  |  |  |  |  |  |  |  |  |  |  |  |  |  |  |  |  |  |  |  |  |  |  |  |  |  |  |  |  |  |  | *mlc-1* | Myosin Light Chain |
|  |  |  |  |  |  |  |  |  |  |  |  |  |  |  |  |  |  |  |  |  |  |  |  |  |  |  |  |  |  |  |  |  |  |  |  |  |  | *emb-9* | abnormal EMBroygenesis |
|  |  |  |  |  |  |  |  |  |  |  |  |  |  |  |  |  |  |  |  |  |  |  |  |  |  |  |  |  |  |  |  |  |  |  |  |  |  | *let-2* | LEThal |
|  |  |  |  |  |  |  |  |  |  |  |  |  |  |  |  |  |  |  |  |  |  |  |  |  |  |  |  |  |  |  |  |  |  |  |  |  |  | *ost-1* | OSTeonectin (SPARC) related |
|  |  |  |  |  |  |  |  |  |  |  |  |  |  |  |  |  |  |  |  |  |  |  |  |  |  |  |  |  |  |  |  |  |  |  |  |  |  | T25F10.6 |  |
|  |  |  |  |  |  |  |  |  |  |  |  |  |  |  |  |  |  |  |  |  |  |  |  |  |  |  |  |  |  |  |  |  |  |  |  |  |  | *mup-2* | MUscle Positioning |
|  |  |  |  |  |  |  |  |  |  |  |  |  |  |  |  |  |  |  |  |  |  |  |  |  |  |  |  |  |  |  |  |  |  |  |  |  |  | T13C2.3 |  |
|  |  |  |  |  |  |  |  |  |  |  |  |  |  |  |  |  |  |  |  |  |  |  |  |  |  |  |  |  |  |  |  |  |  |  |  |  |  | *myo-3* | MYOsin heavy chain structural genes |
|  |  |  |  |  |  |  |  |  |  |  |  |  |  |  |  |  |  |  |  |  |  |  |  |  |  |  |  |  |  |  |  |  |  |  |  |  |  | *unc-95* | UNCoordinated |
|  |  |  |  |  |  |  |  |  |  |  |  |  |  |  |  |  |  |  |  |  |  |  |  |  |  |  |  |  |  |  |  |  |  |  |  |  |  | *unc-54* | UNCoordinated |
|  |  |  |  |  |  |  |  |  |  |  |  |  |  |  |  |  |  |  |  |  |  |  |  |  |  |  |  |  |  |  |  |  |  |  |  |  |  | *unc-15* | UNCoordinated |
|  |  |  |  |  |  |  |  |  |  |  |  |  |  |  |  |  |  |  |  |  |  |  |  |  |  |  |  |  |  |  |  |  |  |  |  |  |  | F21H7.5 |  |
|  |  |  |  |  |  |  |  |  |  |  |  |  |  |  |  |  |  |  |  |  |  |  |  |  |  |  |  |  |  |  |  |  |  |  |  |  |  | F42E11.3 |  |
|  |  |  |  |  |  |  |  |  |  |  |  |  |  |  |  |  |  |  |  |  |  |  |  |  |  |  |  |  |  |  |  |  |  |  |  |  |  | *let-756* | LEThal |
|  |  |  |  |  |  |  |  |  |  |  |  |  |  |  |  |  |  |  |  |  |  |  |  |  |  |  |  |  |  |  |  |  |  |  |  |  |  | W02B8.1 |  |
|  |  |  |  |  |  |  |  |  |  |  |  |  |  |  |  |  |  |  |  |  |  |  |  |  |  |  |  |  |  |  |  |  |  |  |  |  |  | F29B9.8 |  |
|  |  |  |  |  |  |  |  |  |  |  |  |  |  |  |  |  |  |  |  |  |  |  |  |  |  |  |  |  |  |  |  |  |  |  |  |  |  | F21C10.7 |  |
|  |  |  |  |  |  |  |  |  |  |  |  |  |  |  |  |  |  |  |  |  |  |  |  |  |  |  |  |  |  |  |  |  |  |  |  |  |  | *tag-241* | Temporarily Assigned Gene name |
|  |  |  |  |  |  |  |  |  |  |  |  |  |  |  |  |  |  |  |  |  |  |  |  |  |  |  |  |  |  |  |  |  |  |  |  |  |  | C32E12.4 |  |
|  |  |  |  |  |  |  |  |  |  |  |  |  |  |  |  |  |  |  |  |  |  |  |  |  |  |  |  |  |  |  |  |  |  |  |  |  |  | F46C5.6 |  |
|  |  |  |  |  |  |  |  |  |  |  |  |  |  |  |  |  |  |  |  |  |  |  |  |  |  |  |  |  |  |  |  |  |  |  |  |  |  | T12D8.9 |  |
|  |  |  |  |  |  |  |  |  |  |  |  |  |  |  |  |  |  |  |  |  |  |  |  |  |  |  |  |  |  |  |  |  |  |  |  |  |  | *pqn-62* | Prion-like-(Q/N-rich)-domain-bearing protein |
|  |  |  |  |  |  |  |  |  |  |  |  |  |  |  |  |  |  |  |  |  |  |  |  |  |  |  |  |  |  |  |  |  |  |  |  |  |  | *glb-8* | GLoBin related |
|  |  |  |  |  |  |  |  |  |  |  |  |  |  |  |  |  |  |  |  |  |  |  |  |  |  |  |  |  |  |  |  |  |  |  |  |  |  | C03H5.5 |  |
|  |  |  |  |  |  |  |  |  |  |  |  |  |  |  |  |  |  |  |  |  |  |  |  |  |  |  |  |  |  |  |  |  |  |  |  |  |  | T19B10.3 |  |
|  |  |  |  |  |  |  |  |  |  |  |  |  |  |  |  |  |  |  |  |  |  |  |  |  |  |  |  |  |  |  |  |  |  |  |  |  |  | *nas-39* | Nematode AStacin protease |
|  |  |  |  |  |  |  |  |  |  |  |  |  |  |  |  |  |  |  |  |  |  |  |  |  |  |  |  |  |  |  |  |  |  |  |  |  |  | F39G3.2 |  |
|  |  |  |  |  |  |  |  |  |  |  |  |  |  |  |  |  |  |  |  |  |  |  |  |  |  |  |  |  |  |  |  |  |  |  |  |  |  | *anmt-3* | Amine N-MethylTransferase |
|  |  |  |  |  |  |  |  |  |  |  |  |  |  |  |  |  |  |  |  |  |  |  |  |  |  |  |  |  |  |  |  |  |  |  |  |  |  | *spp-15* | SaPosin-like Protein family |
|  |  |  |  |  |  |  |  |  |  |  |  |  |  |  |  |  |  |  |  |  |  |  |  |  |  |  |  |  |  |  |  |  |  |  |  |  |  | *unc-98* | UNCoordinated |
|  |  |  |  |  |  |  |  |  |  |  |  |  |  |  |  |  |  |  |  |  |  |  |  |  |  |  |  |  |  |  |  |  |  |  |  |  |  | F53A3.1 |  |
|  |  |  |  |  |  |  |  |  |  |  |  |  |  |  |  |  |  |  |  |  |  |  |  |  |  |  |  |  |  |  |  |  |  |  |  |  |  | M02D8.1 |  |
|  |  |  |  |  |  |  |  |  |  |  |  |  |  |  |  |  |  |  |  |  |  |  |  |  |  |  |  |  |  |  |  |  |  |  |  |  |  | K07E8.6 |  |
|  |  |  |  |  |  |  |  |  |  |  |  |  |  |  |  |  |  |  |  |  |  |  |  |  |  |  |  |  |  |  |  |  |  |  |  |  |  | *anmt-1* | Amine N-MethylTransferase |
|  |  |  |  |  |  |  |  |  |  |  |  |  |  |  |  |  |  |  |  |  |  |  |  |  |  |  |  |  |  |  |  |  |  |  |  |  |  | *let-268* | LEThal |
|  |  |  |  |  |  |  |  |  |  |  |  |  |  |  |  |  |  |  |  |  |  |  |  |  |  |  |  |  |  |  |  |  |  |  |  |  |  | *icl-1* | IsoCitrate Lyase homolog |
|  |  |  |  |  |  |  |  |  |  |  |  |  |  |  |  |  |  |  |  |  |  |  |  |  |  |  |  |  |  |  |  |  |  |  |  |  |  | *pat-9* | Paralysed Arrest at Two-fold |
|  |  |  |  |  |  |  |  |  |  |  |  |  |  |  |  |  |  |  |  |  |  |  |  |  |  |  |  |  |  |  |  |  |  |  |  |  |  | T05A7.11 |  |
|  |  |  |  |  |  |  |  |  |  |  |  |  |  |  |  |  |  |  |  |  |  |  |  |  |  |  |  |  |  |  |  |  |  |  |  |  |  | *gta-1* | GABA TransAminase family |
|  |  |  |  |  |  |  |  |  |  |  |  |  |  |  |  |  |  |  |  |  |  |  |  |  |  |  |  |  |  |  |  |  |  |  |  |  |  | *unc-82* | UNCoordinated |
|  |  |  |  |  |  |  |  |  |  |  |  |  |  |  |  |  |  |  |  |  |  |  |  |  |  |  |  |  |  |  |  |  |  |  |  |  |  | *unc-89* | UNCoordinated |
|  |  |  |  |  |  |  |  |  |  |  |  |  |  |  |  |  |  |  |  |  |  |  |  |  |  |  |  |  |  |  |  |  |  |  |  |  |  | *set-18* | SET (trithorax/polycomb) domain containing |
|  |  |  |  |  |  |  |  |  |  |  |  |  |  |  |  |  |  |  |  |  |  |  |  |  |  |  |  |  |  |  |  |  |  |  |  |  |  | T05G5.1 |  |
|  |  |  |  |  |  |  |  |  |  |  |  |  |  |  |  |  |  |  |  |  |  |  |  |  |  |  |  |  |  |  |  |  |  |  |  |  |  | *hlh-1* | Helix Loop Helix |
|  |  |  |  |  |  |  |  |  |  |  |  |  |  |  |  |  |  |  |  |  |  |  |  |  |  |  |  |  |  |  |  |  |  |  |  |  |  | C39D10.3 |  |
|  |  |  |  |  |  |  |  |  |  |  |  |  |  |  |  |  |  |  |  |  |  |  |  |  |  |  |  |  |  |  |  |  |  |  |  |  |  | *upb-1* | UreidoPropionase Beta |
|  |  |  |  |  |  |  |  |  |  |  |  |  |  |  |  |  |  |  |  |  |  |  |  |  |  |  |  |  |  |  |  |  |  |  |  |  |  | R02F2.2 |  |
|  |  |  |  |  |  |  |  |  |  |  |  |  |  |  |  |  |  |  |  |  |  |  |  |  |  |  |  |  |  |  |  |  |  |  |  |  |  | *pqn-42* | Prion-like-(Q/N-rich)-domain-bearing protein |
|  |  |  |  |  |  |  |  |  |  |  |  |  |  |  |  |  |  |  |  |  |  |  |  |  |  |  |  |  |  |  |  |  |  |  |  |  |  | *tnt-3* | TropoNin T |
|  |  |  |  |  |  |  |  |  |  |  |  |  |  |  |  |  |  |  |  |  |  |  |  |  |  |  |  |  |  |  |  |  |  |  |  |  |  | *math-4* | MATH (meprin-associated Traf homology) domain containing |
|  |  |  |  |  |  |  |  |  |  |  |  |  |  |  |  |  |  |  |  |  |  |  |  |  |  |  |  |  |  |  |  |  |  |  |  |  |  | *spon-1* | SPONdin (extracellular matrix glycoprotein) family |
|  |  |  |  |  |  |  |  |  |  |  |  |  |  |  |  |  |  |  |  |  |  |  |  |  |  |  |  |  |  |  |  |  |  |  |  |  |  | C05D9.3 |  |
|  |  |  |  |  |  |  |  |  |  |  |  |  |  |  |  |  |  |  |  |  |  |  |  |  |  |  |  |  |  |  |  |  |  |  |  |  |  | R03E9.2 |  |
|  |  |  |  |  |  |  |  |  |  |  |  |  |  |  |  |  |  |  |  |  |  |  |  |  |  |  |  |  |  |  |  |  |  |  |  |  |  | *dhp-2* | DiHydroPyrimidinase |
|  |  |  |  |  |  |  |  |  |  |  |  |  |  |  |  |  |  |  |  |  |  |  |  |  |  |  |  |  |  |  |  |  |  |  |  |  |  | *spp-10* | SaPosin-like Protein family |
|  |  |  |  |  |  |  |  |  |  |  |  |  |  |  |  |  |  |  |  |  |  |  |  |  |  |  |  |  |  |  |  |  |  |  |  |  |  | *gana-1* | GAlactosidase/N-Acetylgalactosaminidase |
|  |  |  |  |  |  |  |  |  |  |  |  |  |  |  |  |  |  |  |  |  |  |  |  |  |  |  |  |  |  |  |  |  |  |  |  |  |  | *lev-11* | LEVamisole resistant |
|  |  |  |  |  |  |  |  |  |  |  |  |  |  |  |  |  |  |  |  |  |  |  |  |  |  |  |  |  |  |  |  |  |  |  |  |  |  | *pitr-1* | PIT (mammalian phosphate transporter) Related |
|  |  |  |  |  |  |  |  |  |  |  |  |  |  |  |  |  |  |  |  |  |  |  |  |  |  |  |  |  |  |  |  |  |  |  |  |  |  | *spv-1* | Spermathecal Physiology Variant |
|  |  |  |  |  |  |  |  |  |  |  |  |  |  |  |  |  |  |  |  |  |  |  |  |  |  |  |  |  |  |  |  |  |  |  |  |  |  | T13H5.1 |  |
|  |  |  |  |  |  |  |  |  |  |  |  |  |  |  |  |  |  |  |  |  |  |  |  |  |  |  |  |  |  |  |  |  |  |  |  |  |  | W05F2.4 |  |
|  |  |  |  |  |  |  |  |  |  |  |  |  |  |  |  |  |  |  |  |  |  |  |  |  |  |  |  |  |  |  |  |  |  |  |  |  |  | *nhl-1* | NHL (ring finger b-box coiled coil) domain containing |
|  |  |  |  |  |  |  |  |  |  |  |  |  |  |  |  |  |  |  |  |  |  |  |  |  |  |  |  |  |  |  |  |  |  |  |  |  |  | R06A10.4 |  |
|  |  |  |  |  |  |  |  |  |  |  |  |  |  |  |  |  |  |  |  |  |  |  |  |  |  |  |  |  |  |  |  |  |  |  |  |  |  | *cor-1* | CORonin |
|  |  |  |  |  |  |  |  |  |  |  |  |  |  |  |  |  |  |  |  |  |  |  |  |  |  |  |  |  |  |  |  |  |  |  |  |  |  | *nhr-69* | Nuclear Hormone Receptor family |
|  |  |  |  |  |  |  |  |  |  |  |  |  |  |  |  |  |  |  |  |  |  |  |  |  |  |  |  |  |  |  |  |  |  |  |  |  |  | *fkb-4* | FK506-Binding protein family |
|  |  |  |  |  |  |  |  |  |  |  |  |  |  |  |  |  |  |  |  |  |  |  |  |  |  |  |  |  |  |  |  |  |  |  |  |  |  | K11D12.7 |  |
|  |  |  |  |  |  |  |  |  |  |  |  |  |  |  |  |  |  |  |  |  |  |  |  |  |  |  |  |  |  |  |  |  |  |  |  |  |  | *mlp-1* | MLP/CRP family (Muscle LIM Protein/Cysteine-rich Protein) |
|  |  |  |  |  |  |  |  |  |  |  |  |  |  |  |  |  |  |  |  |  |  |  |  |  |  |  |  |  |  |  |  |  |  |  |  |  |  | ZC247.2 |  |
|  |  |  |  |  |  |  |  |  |  |  |  |  |  |  |  |  |  |  |  |  |  |  |  |  |  |  |  |  |  |  |  |  |  |  |  |  |  | *unc-120* | UNCoordinated |
|  |  |  |  |  |  |  |  |  |  |  |  |  |  |  |  |  |  |  |  |  |  |  |  |  |  |  |  |  |  |  |  |  |  |  |  |  |  | *dys-1* | DYStrophin related |
|  |  |  |  |  |  |  |  |  |  |  |  |  |  |  |  |  |  |  |  |  |  |  |  |  |  |  |  |  |  |  |  |  |  |  |  |  |  | *fkb-5* | FK506-Binding protein family |
|  |  |  |  |  |  |  |  |  |  |  |  |  |  |  |  |  |  |  |  |  |  |  |  |  |  |  |  |  |  |  |  |  |  |  |  |  |  | *phy-2* | Proline HYdroxylase |
|  |  |  |  |  |  |  |  |  |  |  |  |  |  |  |  |  |  |  |  |  |  |  |  |  |  |  |  |  |  |  |  |  |  |  |  |  |  | Y59C2A.1 |  |
|  |  |  |  |  |  |  |  |  |  |  |  |  |  |  |  |  |  |  |  |  |  |  |  |  |  |  |  |  |  |  |  |  |  |  |  |  |  | *snf-11* | Sodium: Neurotransmitter symporter Family |
|  |  |  |  |  |  |  |  |  |  |  |  |  |  |  |  |  |  |  |  |  |  |  |  |  |  |  |  |  |  |  |  |  |  |  |  |  |  | *act-3* | ACTin |
|  |  |  |  |  |  |  |  |  |  |  |  |  |  |  |  |  |  |  |  |  |  |  |  |  |  |  |  |  |  |  |  |  |  |  |  |  |  | K08H10.2 |  |
|  |  |  |  |  |  |  |  |  |  |  |  |  |  |  |  |  |  |  |  |  |  |  |  |  |  |  |  |  |  |  |  |  |  |  |  |  |  | *him-4* | High Incidence of Males (increased X chromosome loss) |
|  |  |  |  |  |  |  |  |  |  |  |  |  |  |  |  |  |  |  |  |  |  |  |  |  |  |  |  |  |  |  |  |  |  |  |  |  |  | F11F1.5 |  |
|  |  |  |  |  |  |  |  |  |  |  |  |  |  |  |  |  |  |  |  |  |  |  |  |  |  |  |  |  |  |  |  |  |  |  |  |  |  | *fbxc-29* | F-box C protein |
|  |  |  |  |  |  |  |  |  |  |  |  |  |  |  |  |  |  |  |  |  |  |  |  |  |  |  |  |  |  |  |  |  |  |  |  |  |  | C32D5.4 |  |
|  |  |  |  |  |  |  |  |  |  |  |  |  |  |  |  |  |  |  |  |  |  |  |  |  |  |  |  |  |  |  |  |  |  |  |  |  |  | T02H6.6 |  |
|  |  |  |  |  |  |  |  |  |  |  |  |  |  |  |  |  |  |  |  |  |  |  |  |  |  |  |  |  |  |  |  |  |  |  |  |  |  | R102.5 |  |
|  |  |  |  |  |  |  |  |  |  |  |  |  |  |  |  |  |  |  |  |  |  |  |  |  |  |  |  |  |  |  |  |  |  |  |  |  |  | F19B6.3 |  |
|  |  |  |  |  |  |  |  |  |  |  |  |  |  |  |  |  |  |  |  |  |  |  |  |  |  |  |  |  |  |  |  |  |  |  |  |  |  | T20F5.7 |  |
|  |  |  |  |  |  |  |  |  |  |  |  |  |  |  |  |  |  |  |  |  |  |  |  |  |  |  |  |  |  |  |  |  |  |  |  |  |  | F10E7.1 |  |
|  |  |  |  |  |  |  |  |  |  |  |  |  |  |  |  |  |  |  |  |  |  |  |  |  |  |  |  |  |  |  |  |  |  |  |  |  |  | *cln-3.2* | human CLN (neuronal ceroid lipofuscinosis) related |
|  |  |  |  |  |  |  |  |  |  |  |  |  |  |  |  |  |  |  |  |  |  |  |  |  |  |  |  |  |  |  |  |  |  |  |  |  |  | *srz-64* | Serpentine Receptor, class Z |
|  |  |  |  |  |  |  |  |  |  |  |  |  |  |  |  |  |  |  |  |  |  |  |  |  |  |  |  |  |  |  |  |  |  |  |  |  |  | F46F2.9 |  |
|  |  |  |  |  |  |  |  |  |  |  |  |  |  |  |  |  |  |  |  |  |  |  |  |  |  |  |  |  |  |  |  |  |  |  |  |  |  | F44E5.2 |  |
|  |  |  |  |  |  |  |  |  |  |  |  |  |  |  |  |  |  |  |  |  |  |  |  |  |  |  |  |  |  |  |  |  |  |  |  |  |  | K12F2.4 |  |
|  |  |  |  |  |  |  |  |  |  |  |  |  |  |  |  |  |  |  |  |  |  |  |  |  |  |  |  |  |  |  |  |  |  |  |  |  |  | *tag-131* | Temporarily Assigned Gene name |
|  |  |  |  |  |  |  |  |  |  |  |  |  |  |  |  |  |  |  |  |  |  |  |  |  |  |  |  |  |  |  |  |  |  |  |  |  |  | *flap-1* | FLi1-Associated Protein homolog |
|  |  |  |  |  |  |  |  |  |  |  |  |  |  |  |  |  |  |  |  |  |  |  |  |  |  |  |  |  |  |  |  |  |  |  |  |  |  | ZK945.4 |  |
|  |  |  |  |  |  |  |  |  |  |  |  |  |  |  |  |  |  |  |  |  |  |  |  |  |  |  |  |  |  |  |  |  |  |  |  |  |  | *tag-348* | Temporarily Assigned Gene name |
|  |  |  |  |  |  |  |  |  |  |  |  |  |  |  |  |  |  |  |  |  |  |  |  |  |  |  |  |  |  |  |  |  |  |  |  |  |  | ZK177.8 |  |
|  |  |  |  |  |  |  |  |  |  |  |  |  |  |  |  |  |  |  |  |  |  |  |  |  |  |  |  |  |  |  |  |  |  |  |  |  |  | Y42H9B.3 |  |
|  |  |  |  |  |  |  |  |  |  |  |  |  |  |  |  |  |  |  |  |  |  |  |  |  |  |  |  |  |  |  |  |  |  |  |  |  |  | *nas-28* | Nematode AStacin protease |
|  |  |  |  |  |  |  |  |  |  |  |  |  |  |  |  |  |  |  |  |  |  |  |  |  |  |  |  |  |  |  |  |  |  |  |  |  |  | *pyk-2* | PYruvate Kinase |
|  |  |  |  |  |  |  |  |  |  |  |  |  |  |  |  |  |  |  |  |  |  |  |  |  |  |  |  |  |  |  |  |  |  |  |  |  |  | Y57A10A.10 |  |
|  |  |  |  |  |  |  |  |  |  |  |  |  |  |  |  |  |  |  |  |  |  |  |  |  |  |  |  |  |  |  |  |  |  |  |  |  |  | F19C7.3 |  |
|  |  |  |  |  |  |  |  |  |  |  |  |  |  |  |  |  |  |  |  |  |  |  |  |  |  |  |  |  |  |  |  |  |  |  |  |  |  | Y37E3.11 |  |
|  |  |  |  |  |  |  |  |  |  |  |  |  |  |  |  |  |  |  |  |  |  |  |  |  |  |  |  |  |  |  |  |  |  |  |  |  |  | C23G10.5 |  |
|  |  |  |  |  |  |  |  |  |  |  |  |  |  |  |  |  |  |  |  |  |  |  |  |  |  |  |  |  |  |  |  |  |  |  |  |  |  | F56F11.2 |  |
|  |  |  |  |  |  |  |  |  |  |  |  |  |  |  |  |  |  |  |  |  |  |  |  |  |  |  |  |  |  |  |  |  |  |  |  |  |  | *pxn-2* | PeroXidasiN (Drosophila peroxidase) homolog |
|  |  |  |  |  |  |  |  |  |  |  |  |  |  |  |  |  |  |  |  |  |  |  |  |  |  |  |  |  |  |  |  |  |  |  |  |  |  | Y73F8A.26 |  |
|  |  |  |  |  |  |  |  |  |  |  |  |  |  |  |  |  |  |  |  |  |  |  |  |  |  |  |  |  |  |  |  |  |  |  |  |  |  | *nid-1* | NIDogen (basement membrane protein |
|  |  |  |  |  |  |  |  |  |  |  |  |  |  |  |  |  |  |  |  |  |  |  |  |  |  |  |  |  |  |  |  |  |  |  |  |  |  | *nrf-5* | Nose Resistant to Fluoxetine |
|  |  |  |  |  |  |  |  |  |  |  |  |  |  |  |  |  |  |  |  |  |  |  |  |  |  |  |  |  |  |  |  |  |  |  |  |  |  | *cysl-3* | CYsteine Synthase Like |
|  |  |  |  |  |  |  |  |  |  |  |  |  |  |  |  |  |  |  |  |  |  |  |  |  |  |  |  |  |  |  |  |  |  |  |  |  |  | *dmd-3* | DM (Doublesex/MAB-3) Domain family |
|  |  |  |  |  |  |  |  |  |  |  |  |  |  |  |  |  |  |  |  |  |  |  |  |  |  |  |  |  |  |  |  |  |  |  |  |  |  | F07A11.5 |  |
|  |  |  |  |  |  |  |  |  |  |  |  |  |  |  |  |  |  |  |  |  |  |  |  |  |  |  |  |  |  |  |  |  |  |  |  |  |  | *dhs-19* | DeHydrogenases, Short chain |
|  |  |  |  |  |  |  |  |  |  |  |  |  |  |  |  |  |  |  |  |  |  |  |  |  |  |  |  |  |  |  |  |  |  |  |  |  |  | *cpna-2* | CoPiNe domain protein, Atypical |
|  |  |  |  |  |  |  |  |  |  |  |  |  |  |  |  |  |  |  |  |  |  |  |  |  |  |  |  |  |  |  |  |  |  |  |  |  |  | *ttn-1* | TiTiN family |
|  |  |  |  |  |  |  |  |  |  |  |  |  |  |  |  |  |  |  |  |  |  |  |  |  |  |  |  |  |  |  |  |  |  |  |  |  |  | *insc-1* | INSCuteable (Drososphila asymmetric cell division protein) homolog |
|  |  |  |  |  |  |  |  |  |  |  |  |  |  |  |  |  |  |  |  |  |  |  |  |  |  |  |  |  |  |  |  |  |  |  |  |  |  | ZC373.4 |  |
|  |  |  |  |  |  |  |  |  |  |  |  |  |  |  |  |  |  |  |  |  |  |  |  |  |  |  |  |  |  |  |  |  |  |  |  |  |  | K05C4.3 |  |
|  |  |  |  |  |  |  |  |  |  |  |  |  |  |  |  |  |  |  |  |  |  |  |  |  |  |  |  |  |  |  |  |  |  |  |  |  |  | F44E5.3 |  |
|  |  |  |  |  |  |  |  |  |  |  |  |  |  |  |  |  |  |  |  |  |  |  |  |  |  |  |  |  |  |  |  |  |  |  |  |  |  | C31C9.6 |  |
|  |  |  |  |  |  |  |  |  |  |  |  |  |  |  |  |  |  |  |  |  |  |  |  |  |  |  |  |  |  |  |  |  |  |  |  |  |  | *his-12* | HIStone |
|  |  |  |  |  |  |  |  |  |  |  |  |  |  |  |  |  |  |  |  |  |  |  |  |  |  |  |  |  |  |  |  |  |  |  |  |  |  | Y48G1C.13 |  |
|  |  |  |  |  |  |  |  |  |  |  |  |  |  |  |  |  |  |  |  |  |  |  |  |  |  |  |  |  |  |  |  |  |  |  |  |  |  | Y58A7A.10 |  |
|  |  |  |  |  |  |  |  |  |  |  |  |  |  |  |  |  |  |  |  |  |  |  |  |  |  |  |  |  |  |  |  |  |  |  |  |  |  | *srx-13* | Serpentine Receptor, class X |
|  |  |  |  |  |  |  |  |  |  |  |  |  |  |  |  |  |  |  |  |  |  |  |  |  |  |  |  |  |  |  |  |  |  |  |  |  |  | Y116F11B.13 |  |
|  |  |  |  |  |  |  |  |  |  |  |  |  |  |  |  |  |  |  |  |  |  |  |  |  |  |  |  |  |  |  |  |  |  |  |  |  |  | C27B7.6 |  |
|  |  |  |  |  |  |  |  |  |  |  |  |  |  |  |  |  |  |  |  |  |  |  |  |  |  |  |  |  |  |  |  |  |  |  |  |  |  | R01E6.13 |  |

### Phenotypes enriched

|  |  |  |  |
| --- | --- | --- | --- |
| **Group name** | **Number in cluster** | **Enrichment** | **FDR corrected p** |
| body wall muscle morphology variant | 12 | 27.23 | 6.28e-11 |
| striated muscle morphology variant | 12 | 26.49 | 8.57e-11 |
| muscle system morphology variant | 12 | 21.78 | 1.00e-09 |
| body wall muscle sarcomere morphology variant | 10 | 30.25 | 2.46e-09 |
| paralyzed arrested elongation two fold | 8 | 31.12 | 3.02e-07 |
| two fold arrest | 8 | 31.12 | 3.02e-07 |
| late embryonic arrest | 8 | 17.66 | 3.41e-05 |
| body wall muscle development variant | 5 | 29.17 | 9.15e-04 |
| striated muscle development variant | 5 | 29.17 | 9.15e-04 |
| embryonic arrest | 10 | 7.78 | 1.17e-03 |
| body wall muscle thick filament variant | 5 | 27.23 | 1.30e-03 |
| muscle system development variant | 7 | 12.17 | 2.53e-03 |
| striated muscle morphology variant (RNAi) | 11 | 5.87 | 4.45e-03 |
| body wall muscle morphology variant (RNAi) | 11 | 5.87 | 4.45e-03 |
| muscle system morphology variant (RNAi) | 11 | 5.76 | 5.29e-03 |
| late paralysis arrested elongation two fold | 3 | 61.26 | 9.72e-03 |
| sperm pseudopod variant | 4 | 23.34 | 2.31e-02 |
| organ system development variant | 15 | 3.45 | 3.53e-02 |
| muscle birefringence variant | 4 | 20.42 | 3.81e-02 |

### Anatomy terms enriched

|  |  |  |  |
| --- | --- | --- | --- |
| **Group name** | **Number in cluster** | **Enrichment** | **FDR corrected p** |
| body wall musculature | 65 | 4.84 | 3.77e-23 |
| muscular system | 70 | 3.56 | 9.32e-18 |
| anal depressor muscle | 34 | 6.61 | 6.53e-14 |
| vulval muscle | 38 | 5.31 | 4.89e-13 |
| body muscle cell | 44 | 4.12 | 9.31e-12 |
| alimentary muscle | 38 | 4.32 | 2.17e-10 |
| muscle cell | 49 | 3.35 | 3.53e-10 |
| non-striated muscle | 46 | 3.36 | 1.87e-09 |
| anal sphincter muscle | 16 | 9.14 | 1.56e-07 |
| smooth muscle | 25 | 4.79 | 4.18e-07 |
| hermaphrodite-specific | 50 | 2.46 | 4.06e-06 |
| Sex specific entity | 50 | 2.36 | 1.52e-05 |
| intestinal muscle | 15 | 6.16 | 7.27e-05 |
| Organ system | 84 | 1.69 | 1.74e-04 |
| Function | 84 | 1.68 | 1.84e-04 |
| basal lamina | 4 | 54.45 | 8.36e-04 |
| C. elegans Cell and Anatomy Ontology | 84 | 1.59 | 1.90e-03 |
| GLR | 5 | 19.45 | 9.48e-03 |
| Cell | 69 | 1.64 | 1.14e-02 |

### GO terms enriched

|  |  |  |
| --- | --- | --- |
| **GO term** | **Number of genes** | **FDR-corrected p-value** |
| basement membrane | 7 | 1.4e-06 |
| striated muscle cell differentiation | 14 | 1.7e-06 |
| cellular component morphogenesis | 18 | 9.4e-06 |
| muscle cell development | 13 | 1.3e-05 |
| muscle organ development | 7 | 2.9e-05 |
| extracellular matrix | 10 | 3.1e-05 |
| muscle structure development | 12 | 6.3e-05 |
| striated muscle dense body | 9 | 1.4e-04 |
| contractile fiber | 7 | 1.6e-04 |
| calcium ion binding | 11 | 2.7e-04 |
| non-membrane-bounded organelle | 27 | 8.6e-04 |
| myosin filament organization | 10 | 9.7e-04 |
| sarcomere | 6 | 1.1e-03 |
| anatomical structure formation involved in morphogenesis | 13 | 1.3e-03 |
| cytoplasm | 42 | 3.3e-03 |
| cellular protein complex assembly | 11 | 6.1e-03 |
| M band | 5 | 6.3e-03 |
| extracellular region | 15 | 7.1e-03 |
| organelle part | 30 | 7.5e-03 |
| myofilament | 4 | 1.2e-02 |
| actin cytoskeleton organization | 6 | 1.2e-02 |
| regulation of muscle contraction | 4 | 1.9e-02 |
| protein complex biogenesis | 11 | 2.2e-02 |
| regulation of system process | 4 | 2.5e-02 |
| macromolecular complex assembly | 12 | 2.8e-02 |
| protein complex subunit organization | 13 | 3.8e-02 |
| myofibril assembly | 3 | 4.2e-02 |

### Expression clusters enriched

|  |  |  |  |
| --- | --- | --- | --- |
| **Group name** | **Number in cluster** | **Enrichment** | **FDR corrected p** |
| Genes enriched in muscle cells (0hr muscle dataset). Dissociated myo-3::GFP embryos were cultured for 0 hours before FACS sorting. | 111 | 11.90 | 1.69e-91 |
| Total muscle enriched genes (complete list of non-overlapping genes from the 0hr and 24hr muscle enriched datasets). | 125 | 7.94 | 4.36e-84 |
| Genes enriched in muscle cells (24hr muscle dataset). Dissociated myo-3::GFP embryos were cultured for 24 hours before FACS sorting. | 97 | 8.63 | 5.08e-64 |
| Genes that show selective expression in a subset of cell types vs broadly expressed in many cell types. Correspond to 20% - 57% of enriched\_genes for a given cell type. WBPaper00037950:bodywall-muscle\_larva\_SelectivelyEnriched | 71 | 12.95 | 1.79e-56 |
| Genes enriched in HLH-1 heat shock dataset. | 126 | 4.44 | 1.31e-54 |
| Genes that show selective expression in a subset of cell types vs broadly expressed in many cell types. Correspond to 20% - 57% of enriched\_genes for a given cell type. WBPaper00037950:bodywall-muscle\_embryo\_SelectivelyEnriched | 38 | 17.15 | 6.63e-33 |
| Genes significantly enriched (> 2x, FDR < 5%) in a particular cell-type versus a reference sample of all cells at the same stage. WBPaper00037950:bodywall-muscle\_larva\_enriched | 75 | 5.33 | 2.20e-32 |
| Genes significantly enriched (> 2x, FDR < 5%) in a particular cell-type versus a reference sample of all cells at both embryonic and larval stages. WBPaper00037950:bodywall-muscle\_CoreEnriched | 27 | 18.23 | 1.90e-23 |
| Genes significantly enriched (> 2x, FDR < 5%) in a particular cell-type versus a reference sample of all cells at the same stage. WBPaper00037950:bodywall-muscle\_embryo\_enriched | 39 | 8.09 | 3.11e-21 |
| Genes enriched in body wall muscle AIN-2 miRISCs. Pmyo-3-ain-2-gfp IP was performed in mixed stage worms. | 38 | 5.93 | 5.44e-16 |
| Genes predicted to be downregulated more than 2.0 fold in (AFD+AWB) datasets as compared to unsorted whole embryonic cells dataset. | 38 | 5.90 | 6.57e-16 |
| Expression Pattern Group C, enriched for genes involved in metabolic processes. | 48 | 3.95 | 8.96e-14 |
| TGF- Dauer pathway adult transcriptional targets. Results obtained by comparing the microarray results of the dauer-constitutive mutants daf-7(e1372), daf-7(m62), and daf-1(m40) with dauer-defective mutants daf-3(mgDf90), daf-5(e1386), and daf-7(e1372);daf-3(mgDf90) double mutants at the permissive temperature, 20C, on the first day of adulthood. WBPaper00031040:TGF-beta\_adult\_upregulated | 66 | 2.48 | 3.48e-10 |
| Genes that showed increased expression after treated with 2-deoxy-D-glucose. | 19 | 6.13 | 1.97e-07 |
| WT-Pico Pan-neural Depleted Genes, with genes found multiple times in a single dataset removed (without dups). | 42 | 2.73 | 9.32e-07 |
| Genes downregulated by CYD-1/CDK-4 for more than two fold. | 26 | 3.93 | 1.40e-06 |
| C-lineage related expression profile. WBPaper00025032:cluster\_24 | 9 | 15.64 | 2.63e-06 |
| Germline-enriched and sex-biased expression profile cluster C. | 33 | 3.07 | 4.05e-06 |
| Genes in the top 10% of expression level across the triplicate L3 samples. To generate the top10 and bottom10 gene sets, authors ranked all genes by mean expression array signal intensity across the three replicates, then took the top and bottom deciles (1,841 genes each) to represent genes with high and low expression. | 51 | 2.26 | 6.08e-06 |
| The cluster contains genes that are significantly enriched in L1 muscle. | 43 | 2.43 | 1.48e-05 |
| Transcripts that cycle in LD(light/dark) but not in DD(constant darkness) (pF24<0.02) | 28 | 3.18 | 2.69e-05 |
| Potential PAL-1 target genes. | 13 | 6.60 | 4.15e-05 |
| intestinal (adult); 1.2e-04(1.11x). | 20 | 3.97 | 7.06e-05 |
| Genes up-regulated after 300 um Tannic acid treatment. Fold change > 1.25. | 36 | 2.55 | 7.23e-05 |
| intestinal (adult); 8.3e-05(1.12x). | 20 | 3.97 | 7.32e-05 |
| intestinal (adult); 3.7e-05(1.14x). topomap mount 7; 5.4e-04(1.54x). | 21 | 3.60 | 1.64e-04 |
| intestinal (adult); 4.5e-05(1.13x). topomap mount 7; 5.6e-04(1.54x). | 21 | 3.60 | 1.69e-04 |
| TGF-beta adult-specific up/dauer down | 9 | 8.55 | 4.03e-04 |
| Genes differentially expressed under EtBr treatment and UVC exposure vs under UVC exposure but without EtBr treatment at the -3h timepoint (3 h after the third UVC dose (51h), which is also 3 h after being placed on food). | 54 | 1.87 | 8.05e-04 |
| Proteins that showed increased expression 24h after infection with S. aureus. | 6 | 11.14 | 4.45e-03 |
| Gene significantly up-regulated by treatment with 2.0mM of HuminFeed until young adult stage (3 days), with a minimum fold change in gene expression of 1.25. | 38 | 2.02 | 5.42e-03 |
| Genes differentially expressed in control vs after UVC exposure and EtBr treatment at the 3h timepoint (3 h after the third UVC dose (51h), which is also 3 h after being placed on food). | 72 | 1.53 | 1.01e-02 |
| Genes with expression altered >= 3-fold in dpy-9(e12) mutants. | 57 | 1.66 | 1.14e-02 |
| Genes with no change in hcf-1(-), no change in sir-2.1(O/E) and upregulated in daf-2(-). | 26 | 2.34 | 1.19e-02 |
| Genes differentially expressed under UVC exposure and EtBr treatment vs under EtBr treatment but without UVC exposure at the -3h timepoint (3 h after the third UVC dose (51h), which is also 3 h after being placed on food). | 36 | 1.96 | 1.58e-02 |
| Genes with expression altered >= 3-fold in dpy-10(e128) mutants. | 64 | 1.57 | 1.65e-02 |
| Genes with expression altered >= 3-fold in osm-8(n1518) mutants. | 28 | 2.20 | 1.67e-02 |
| Genes downregulated by fer-1 mutants hc1 and hc24, with > 4 fold change in expression level. | 11 | 4.10 | 2.02e-02 |
| Genome-wide analysis of developmental and sex-regulated gene expression profile. cgc4489\_group\_24 | 12 | 3.46 | 4.30e-02 |
| Jiang M. et al. group 3; 6.0e-04(2.10x). | 6 | 7.10 | 4.33e-02 |
| body wall muscle (adult); 6.4e-04(1.54x). Jiang M. et al. group 3; 7.2e-04(2.07x). ex). cell (adult); 8.9e-04(2.31x). | 6 | 7.00 | 4.62e-02 |

### Motifs enriched

|  |  |  |  |  |  |
| --- | --- | --- | --- | --- | --- |
| **Motif** | **Logo** | **Possible orthologs** | **Number of motifs in cluster** | **Enrichment** | **FDR corrected p** |
| pTH10808 |  | ztf-19 | 44 | 6.48 | 3.4e-19 |
| MA0545.1 |  | hlh-1 (0.94) hlh-11 lin-32 hlh-15 hlh-14 | 113 | 1.91 | 3.0e-12 |
| Myf6\_3824 |  | hlh-1 (0.94) hlh-8 (0.87) K02D7.2 (0.82) hlh-15 hlh-2 ces-1 | 71 | 2.36 | 8.0e-10 |
| MA0543.1 |  | daf-8 eor-1 | 108 | 1.78 | 1.9e-09 |
| NDF1\_f1 |  | hlh-8 (0.87) hlh-32 hlh-15 ngn-1 hlh-12 | 88 | 2.01 | 2.3e-09 |
| Meis3\_2 |  | lin-39 ceh-32 lin-32 | 103 | 1.78 | 1.2e-08 |
| MA0162.2 |  | klf-2 klf-1 ZC328.2 | 45 | 2.98 | 2.4e-08 |
| pTH6071 |  | C46E10.8 C33G8.2 | 74 | 2.02 | 2.1e-07 |
| TFE3\_f1 |  | hlh-30 pax-1 mdl-1 aha-1 | 120 | 1.55 | 2.7e-07 |
| MITF\_f1 |  | hlh-30 mxl-1 | 112 | 1.61 | 2.9e-07 |
| NEUROG2\_2 |  | hlh-8 (0.87) hlh-32 hlh-15 ngn-1 | 70 | 2.06 | 3.5e-07 |
| TBX20\_5 |  | mab-9 tbx-39 tbx-38 tbx-43 | 103 | 1.66 | 4.6e-07 |
| MA0493.1 |  | klf-2 klf-1 | 96 | 1.72 | 4.7e-07 |
| MA0070.1 |  | ceh-20 (0.55) lin-39 ceh-12 | 78 | 1.89 | 8.9e-07 |
| V$E47\_02 |  | lin-32 hlh-2 | 76 | 1.90 | 1.5e-06 |
| Foxj3\_2 |  | lin-31 (0.74) let-381 pha-4 fkh-8 daf-16 fkh-7 | 136 | 1.42 | 1.6e-06 |
| pTH3796 |  | lin-31 (0.74) let-381 fkh-10 C34D1.1 | 101 | 1.63 | 2.4e-06 |
| pTH10037 |  | T22C8.4 (0.52) ref-2 | 87 | 1.75 | 2.7e-06 |
| MA0472.1 |  | ZC328.2 daf-16 | 35 | 2.99 | 2.8e-06 |
| NR2F6\_f1 |  | nhr-239 nhr-62 nhr-2 | 108 | 1.57 | 2.9e-06 |
| ZNF75A\_1 |  | ztf-3 lag-1 F26F4.8 | 113 | 1.54 | 3.0e-06 |
| TCF4\_2 |  | hlh-2 ztf-6 | 104 | 1.59 | 3.7e-06 |
| MA0508.1 |  | blmp-1 | 104 | 1.59 | 3.9e-06 |
| pTH5065 |  | ref-1 lin-22 hlh-30 hlh-26 mxl-1 mxl-2 aha-1 C27D6.4 | 82 | 1.78 | 4.7e-06 |
| pTH3469 |  | nhr-69 (0.93) nhr-19 (0.78) nhr-10 nhr-213 nhr-2 | 84 | 1.75 | 5.3e-06 |
| V$SP1\_Q6 |  | klf-2 klf-1 ZC328.2 | 67 | 1.95 | 6.4e-06 |
| pTH3037 |  | hlh-1 (0.94) hlh-15 | 70 | 1.90 | 6.4e-06 |
| pTH2820 |  | ZC328.2 | 45 | 2.43 | 6.8e-06 |
| PURA\_f1 |  | klf-2 Y53H1A.2 plp-2 | 92 | 1.66 | 9.2e-06 |
| pTH10772 |  | ceh-52 | 60 | 2.04 | 1.0e-05 |
| V$FOXO3\_01 |  | lin-31 (0.74) let-381 fkh-8 daf-16 fkh-7 fkh-10 | 95 | 1.63 | 1.0e-05 |
| MA0035.3 |  | elt-1 | 106 | 1.55 | 1.1e-05 |
| GATA5\_f1 |  | nhr-10 elt-1 nhr-100 nhr-7 | 109 | 1.53 | 1.2e-05 |
| pTH10623 |  | scrt-1 | 106 | 1.54 | 1.2e-05 |
| HES1\_f1 |  | lin-22 | 99 | 1.59 | 1.3e-05 |
| pTH9394 |  | klf-2 klf-1 sptf-3 | 97 | 1.60 | 1.3e-05 |
| So\_Cell\_FBgn0003460 |  | ceh-32 skn-1 ztf-28 | 114 | 1.49 | 1.6e-05 |
| pTH10722 |  | ref-2 eor-1 egrh-3 | 52 | 2.16 | 1.7e-05 |
| Pknox2\_3077 |  | ceh-32 | 101 | 1.56 | 1.8e-05 |
| V$FAC1\_01 |  | gei-8 | 98 | 1.59 | 1.8e-05 |
| pTH9924 |  | nhr-46 | 126 | 1.42 | 1.9e-05 |
| MA0139.1 |  | Y5F2A.4 F58G1.2 | 79 | 1.75 | 1.9e-05 |
| V$AREB6\_04 |  | ztf-6 C34D1.1 gei-11 | 111 | 1.49 | 2.5e-05 |
| V$GATA1\_04 |  | elt-1 | 97 | 1.58 | 2.6e-05 |
| MEIS3\_1 |  | ceh-20 (0.55) ceh-32 ces-1 F55C5.11 | 104 | 1.53 | 2.7e-05 |
| pTH6429 |  | F13H6.1 nhr-177 nhr-86 nhr-2 | 92 | 1.62 | 2.7e-05 |
| HeLa-S3\_RFX5\_Stanford |  | daf-19 | 89 | 1.63 | 3.6e-05 |
| V$GATA3\_01 |  | elt-1 | 100 | 1.55 | 3.6e-05 |
| pTH8981 |  | lin-1 ztf-14 ZC328.2 ref-2 pax-3 | 85 | 1.65 | 4.5e-05 |
| ARNT2\_si |  | C46E10.9 aha-1 | 85 | 1.65 | 5.2e-05 |
| pTH9165 |  | ztf-27 | 101 | 1.53 | 5.6e-05 |
| pTH6641 |  | lin-31 (0.74) | 96 | 1.56 | 5.7e-05 |
| V$HOX13\_01 |  | lin-39 | 104 | 1.50 | 6.9e-05 |
| pTH9957 |  | fkh-9 daf-16 irx-1 | 97 | 1.54 | 9.2e-05 |
| V$CDXA\_01 |  | ceh-13 | 29 | 2.87 | 9.3e-05 |
| GATA3\_2 |  | elt-1 end-3 | 96 | 1.54 | 9.6e-05 |
| pTH1292 |  | pzf-1 (-0.51) ceh-24 | 101 | 1.51 | 9.6e-05 |
| pTH9043 |  | sem-2 | 117 | 1.42 | 9.7e-05 |
| HXA7\_f1 |  | lin-39 elt-1 | 100 | 1.51 | 1.0e-04 |
| So\_SOLEXA\_FBgn0003460 |  | elt-3 (0.76) ceh-32 elt-7 elt-1 elt-6 ceh-34 | 115 | 1.43 | 1.0e-04 |
| ZBT7A\_f1 |  | klf-1 ZC328.2 | 85 | 1.62 | 1.1e-04 |
| ftz-f1\_FlyReg\_FBgn0001078 |  | lin-31 (0.74) nhr-68 F55C5.11 | 104 | 1.49 | 1.1e-04 |
| ZN384\_f1 |  | K11D2.4 lin-29 | 160 | 1.24 | 1.2e-04 |
| K562\_ZBTB7A\_HudsonAlpha |  | ZC328.2 | 84 | 1.62 | 1.2e-04 |
| FOXC2\_3 |  | lin-31 (0.74) let-381 | 130 | 1.36 | 1.2e-04 |
| pTH2846 |  | lin-31 (0.74) | 121 | 1.40 | 1.2e-04 |
| Six4\_2860 |  | ceh-32 | 69 | 1.76 | 1.3e-04 |
| CG31670\_SOLEXA\_5\_FBgn0031375 |  | F21A9.2 CELE\_Y38H8A.5 | 128 | 1.36 | 1.4e-04 |
| Sox4 |  | sox-4 pop-1 | 78 | 1.66 | 1.5e-04 |
| MA0244.1 |  | C48E7.11 | 81 | 1.63 | 1.7e-04 |
| pTH9132 |  | php-3 (0.62) pal-1 (0.55) lin-39 ceh-13 ceh-24 T27F2.4 D1005.3 | 98 | 1.51 | 1.7e-04 |
| MYB\_f1 |  | D1081.8 | 37 | 2.38 | 1.7e-04 |
| pTH5437 |  | ceh-34 | 82 | 1.62 | 1.9e-04 |
| V$AREB6\_02 |  | ztf-6 | 94 | 1.53 | 1.9e-04 |
| pTH9135 |  | pop-1 | 133 | 1.33 | 2.1e-04 |
| pTH2193 |  | nhr-213 nhr-15 nhr-239 nhr-2 | 89 | 1.56 | 2.2e-04 |
| PBDE\_GATA1\_UCD |  | elt-1 | 107 | 1.45 | 2.2e-04 |
| pTH8996 |  | hlh-8 (0.87) sma-4 | 102 | 1.48 | 2.2e-04 |
| MA0262.1 |  | mab-3 (0.55) | 124 | 1.37 | 2.3e-04 |
| pTH5891 |  | nhr-49 | 80 | 1.62 | 2.4e-04 |
| MA0163.1 |  | Y53H1A.2 D1081.8 | 86 | 1.58 | 2.5e-04 |
| SRP000217\_Sox2 |  | sox-4 ceh-18 | 88 | 1.55 | 2.8e-04 |
| pTH8411 |  | tbx-39 | 108 | 1.43 | 3.2e-04 |
| HLH27 |  | lin-22 hlh-27 hlh-28 | 81 | 1.60 | 3.3e-04 |
| pTH9044 |  | bed-3 nhr-177 F26F4.8 mbr-1 | 69 | 1.71 | 3.3e-04 |
| pTH8991 |  | cey-3 | 58 | 1.84 | 3.4e-04 |
| pTH5914 |  | attf-1 | 127 | 1.34 | 3.5e-04 |
| NR4A2\_si |  | nhr-6 nhr-71 nhr-10 nhr-213 nhr-2 nhr-68 Y67D8A.3 | 104 | 1.45 | 3.7e-04 |
| pTH9163 |  | nhr-3 | 43 | 2.11 | 3.8e-04 |
| pTH10647 |  | nhr-232 | 97 | 1.49 | 3.8e-04 |
| dsx-F\_FlyReg\_FBgn0000504 |  | dmd-4 sox-4 | 92 | 1.52 | 3.9e-04 |
| T-47D\_GATA3\_HudsonAlpha |  | elt-1 | 98 | 1.48 | 4.0e-04 |
| pTH5270 |  | hlh-32 hlh-16 ngn-1 | 76 | 1.63 | 4.0e-04 |
| I$SN\_02 |  | K02D7.2 (0.82) C24A1.2 | 95 | 1.50 | 4.1e-04 |
| Tcf3\_3787 |  | pop-1 | 126 | 1.34 | 4.1e-04 |
| pTH9335 |  | mel-28 | 139 | 1.29 | 4.3e-04 |
| pTH6003 |  | nhr-182 nhr-134 | 113 | 1.40 | 4.5e-04 |
| V$TAXCREB\_02 |  | crh-1 | 86 | 1.55 | 4.5e-04 |
| pTH9709 |  | die-1 | 118 | 1.38 | 4.7e-04 |
| Six1\_0935 |  | ceh-32 | 78 | 1.61 | 4.9e-04 |
| HNF4A\_6 |  | nhr-62 nhr-2 | 105 | 1.43 | 4.9e-04 |
| pTH1001 |  | dnj-17 | 95 | 1.49 | 4.9e-04 |
| MA0451.1 |  | php-3 (0.62) nhr-2 | 93 | 1.50 | 5.3e-04 |
| pTH5928 |  | ceh-34 | 64 | 1.73 | 5.3e-04 |
| FOXC1\_2 |  | lin-31 (0.74) ceh-53 (-0.62) let-381 hmg-12 hmbx-1 Y116A8C.22 | 97 | 1.47 | 5.4e-04 |
| MA0476.1 |  | sknr-1 fos-1 jun-1 F45H11.6 | 44 | 2.05 | 5.6e-04 |
| pTH10629 |  | hif-1 ceh-9 Y5F2A.4 daf-12 ztf-3 | 109 | 1.41 | 5.7e-04 |
| pTH6591 |  | lin-31 (0.74) | 97 | 1.47 | 6.0e-04 |
| Mafk\_3106 |  | F45H11.6 | 78 | 1.60 | 6.0e-04 |
| pTH10777 |  | dmd-3 (0.62) | 93 | 1.49 | 6.1e-04 |
| pTH6486 |  | nhr-145 | 119 | 1.36 | 6.4e-04 |
| V$GATA1\_01 |  | elt-1 | 76 | 1.61 | 6.5e-04 |
| Mv90 |  | mef-2 | 101 | 1.44 | 6.7e-04 |
| pTH10031 |  | mbr-1 | 68 | 1.68 | 6.8e-04 |
| pTH10816 |  | dmd-6 | 117 | 1.37 | 6.9e-04 |
| ISL2\_1 |  | lim-7 ceh-9 ceh-31 | 88 | 1.52 | 7.2e-04 |
| MA0094.2 |  | egl-5 (0.71) cog-1 (0.7) ceh-53 (-0.62) lin-39 ceh-1 ceh-43 ceh-45 lim-7 ceh-14 ceh-2 ceh-36 ceh-12 ceh-31 ceh-23 ceh-16 ceh-30 lim-6 ceh-10 alr-1 T13C5.4 | 109 | 1.40 | 7.2e-04 |
| pTH9242 |  | mel-28 | 127 | 1.33 | 7.3e-04 |
| MA0118.1 |  | ref-2 | 79 | 1.58 | 7.5e-04 |
| MA0135.1 |  | php-3 (0.62) cfi-1 (0.54) lin-39 lim-7 unc-86 | 132 | 1.31 | 7.7e-04 |
| pTH9300 |  | dmd-3 (0.62) C34D1.1 | 108 | 1.41 | 7.8e-04 |
| BARHL2\_3 |  | ceh-1 ceh-43 ceh-14 ceh-31 | 105 | 1.42 | 7.9e-04 |
| MCR\_f1 |  | nhr-255 | 108 | 1.40 | 8.5e-04 |
| YY1\_1 |  | lsy-2 | 100 | 1.44 | 8.6e-04 |
| pTH9247 |  | dmd-3 (0.62) C34D1.1 | 129 | 1.31 | 8.9e-04 |
| pTH9137 |  | nhr-65 | 140 | 1.27 | 1.0e-03 |
| MA0486.1 |  | hsf-1 (0.55) F10B5.3 Y53C10A.3 | 110 | 1.39 | 1.0e-03 |
| CG4854\_SANGER\_10\_FBgn0038766 |  | K11D2.4 | 103 | 1.42 | 1.0e-03 |
| pTH9951 |  | pal-1 (0.55) mex-6 | 133 | 1.29 | 1.1e-03 |
| NKX21\_f1 |  | dsc-1 (0.84) ceh-22 ceh-24 | 88 | 1.50 | 1.2e-03 |
| pTH10823 |  | B0310.2 (0.57) | 96 | 1.45 | 1.2e-03 |
| Six2\_2307 |  | ceh-32 ceh-34 | 63 | 1.69 | 1.2e-03 |
| Caup\_SOLEXA\_FBgn0015919 |  | irx-1 | 62 | 1.70 | 1.2e-03 |
| V$IK2\_01 |  | F26F4.8 | 77 | 1.57 | 1.3e-03 |
| pTH6497 |  | lin-31 (0.74) | 92 | 1.47 | 1.3e-03 |
| K562\_SP2\_HudsonAlpha |  | klf-2 | 82 | 1.53 | 1.3e-03 |
| MA0594.1 |  | php-3 (0.62) lin-39 hbl-1 | 65 | 1.67 | 1.3e-03 |
| CG2052\_SANGER\_2.5\_FBgn0039905 |  | fkh-7 lin-29 mel-28 | 156 | 1.21 | 1.3e-03 |
| pTH9907 |  | nhr-34 | 106 | 1.40 | 1.4e-03 |
| pTH10640 |  | ceh-32 dmd-4 dmd-5 | 63 | 1.68 | 1.5e-03 |
| MA0174.1 |  | php-3 (0.62) lin-39 ceh-24 | 82 | 1.52 | 1.5e-03 |
| pTH10718 |  | egl-43 | 102 | 1.41 | 1.5e-03 |
| Hoxb8\_3780 |  | lin-39 | 80 | 1.53 | 1.6e-03 |
| Gata5\_3768 |  | elt-1 | 70 | 1.61 | 1.7e-03 |
| pTH3998 |  | tbx-39 | 72 | 1.59 | 1.7e-03 |
| V$CEBP\_01 |  | C48E7.11 | 100 | 1.42 | 1.7e-03 |
| LHX2\_f1 |  | ceh-14 alr-1 | 102 | 1.41 | 1.7e-03 |
| MA0161.1 |  | nfi-1 F49E12.6 | 87 | 1.49 | 1.7e-03 |
| pTH10013 |  | nhr-168 | 112 | 1.36 | 1.8e-03 |
| pTH5164 |  | hlh-32 D1081.8 irx-1 | 80 | 1.53 | 1.9e-03 |
| SOX10\_2 |  | sox-4 K11D2.4 egl-13 | 106 | 1.38 | 2.0e-03 |
| Mf28 |  | elt-1 | 85 | 1.49 | 2.0e-03 |
| pTH10650 |  | nhr-153 | 103 | 1.40 | 2.1e-03 |
| VDR\_1 |  | cfi-1 (0.54) gei-3 lim-6 nhr-208 | 78 | 1.54 | 2.1e-03 |
| pTH9244 |  | tbx-39 | 70 | 1.60 | 2.1e-03 |
| SMAD3\_f1 |  | daf-8 | 90 | 1.46 | 2.1e-03 |
| pTH8566 |  | lin-54 | 133 | 1.28 | 2.2e-03 |
| V$CDPCR3\_01 |  | ceh-48 | 102 | 1.40 | 2.3e-03 |
| pTH6508 |  | nhr-36 | 92 | 1.45 | 2.3e-03 |
| V$YY1\_02 |  | lsy-2 | 83 | 1.50 | 2.4e-03 |
| V$XFD3\_01 |  | ceh-20 (0.55) cfi-1 (0.54) let-381 | 134 | 1.27 | 2.4e-03 |
| FOXG1\_2 |  | lin-31 (0.74) | 102 | 1.39 | 2.6e-03 |
| NR2E3\_f1 |  | lin-39 lin-1 nhr-100 | 176 | 1.14 | 2.6e-03 |
| Lhx1\_2240 |  | lim-7 | 85 | 1.48 | 2.7e-03 |
| V$FOXJ2\_02 |  | lin-31 (0.74) | 106 | 1.37 | 2.7e-03 |
| pTH5781 |  | ceh-32 | 96 | 1.42 | 2.9e-03 |
| Tcf7\_0950 |  | pop-1 | 115 | 1.33 | 3.0e-03 |
| pTH5250 |  | C48E7.11 | 109 | 1.36 | 3.0e-03 |
| V$CDP\_01 |  | ceh-48 | 108 | 1.36 | 3.1e-03 |
| pTH3477 |  | daf-16 | 88 | 1.45 | 3.1e-03 |
| pTH9353 |  | ceh-51 | 92 | 1.43 | 3.2e-03 |
| MA0483.1 |  | odd-1 lim-6 | 72 | 1.56 | 3.3e-03 |
| pTH9925 |  | nhr-100 ztf-11 | 129 | 1.28 | 3.4e-03 |
| V$PAX5\_01 |  | pax-2 F45H11.6 | 51 | 1.77 | 3.4e-03 |
| pTH10788 |  | tbx-33 | 118 | 1.32 | 3.5e-03 |
| CENPB\_1 |  | F21D5.4 | 90 | 1.44 | 3.5e-03 |
| TBX3\_f1 |  | tbx-39 | 111 | 1.34 | 3.6e-03 |
| Six3\_1732 |  | ceh-34 | 75 | 1.53 | 3.8e-03 |
| MA0248.1 |  | cog-1 (0.7) lin-39 ceh-1 ceh-43 ceh-19 lim-7 ceh-9 ceh-31 ceh-30 ceh-24 alr-1 ceh-8 | 85 | 1.46 | 3.9e-03 |
| Plagl1\_0972 |  | Y53H1A.2 | 58 | 1.67 | 4.1e-03 |
| TBX2\_f1 |  | tbx-39 ztf-6 | 117 | 1.32 | 4.2e-03 |
| MA0452.2 |  | B0310.2 (0.57) ZK177.3 | 51 | 1.75 | 4.3e-03 |
| Tcf7l2\_3461 |  | ceh-20 (0.55) pop-1 | 108 | 1.35 | 4.4e-03 |
| V$FREAC7\_01 |  | lin-31 (0.74) | 79 | 1.49 | 4.4e-03 |
| Sox8\_1733 |  | gei-3 sox-4 pop-1 C05C9.3 | 118 | 1.31 | 4.5e-03 |
| pTH3751 |  | tbx-39 | 84 | 1.46 | 4.6e-03 |
| Irx3\_1 |  | irx-1 | 151 | 1.21 | 4.6e-03 |
| Hoxd8\_2644 |  | lin-39 | 79 | 1.49 | 4.7e-03 |
| ETS2\_f1 |  | lin-1 | 113 | 1.33 | 4.8e-03 |
| STF1\_f1 |  | nhr-68 | 82 | 1.47 | 4.8e-03 |
| Hr46\_SANGER\_5\_FBgn0000448 |  | nhr-213 nhr-118 | 83 | 1.46 | 4.8e-03 |
| EN1\_4 |  | ceh-16 | 95 | 1.40 | 5.0e-03 |
| pTH7875 |  | mel-28 | 107 | 1.35 | 5.1e-03 |
| MA0474.1 |  | lin-1 C24A1.2 | 106 | 1.35 | 5.1e-03 |
| pTH1049 |  | elt-1 | 86 | 1.44 | 5.2e-03 |
| V$CDPCR1\_01 |  | ceh-48 | 88 | 1.43 | 5.2e-03 |
| pTH5690 |  | ceh-32 | 80 | 1.48 | 5.3e-03 |
| V$PAX5\_02 |  | pax-2 | 97 | 1.39 | 5.4e-03 |
| pTH9480 |  | ces-2 C48E7.11 | 102 | 1.36 | 5.9e-03 |
| MA0027.1 |  | ceh-16 | 80 | 1.47 | 6.0e-03 |
| pTH5714 |  | nhr-239 | 91 | 1.41 | 6.2e-03 |
| pTH5561 |  | nhr-239 | 51 | 1.72 | 6.3e-03 |
| pTH6482 |  | ceh-19 | 86 | 1.43 | 6.4e-03 |
| CG8765\_SANGER\_5\_FBgn0036900 |  | H20J04.3 | 65 | 1.57 | 6.5e-03 |
| Cutl1\_3494 |  | ceh-44 | 82 | 1.45 | 6.6e-03 |
| pTH9189 |  | dmd-3 (0.62) ceh-18 | 115 | 1.31 | 6.6e-03 |
| Dll\_Cell\_FBgn0000157 |  | pal-1 (0.55) lin-39 ceh-43 | 98 | 1.37 | 6.7e-03 |
| Nsy-7 |  | hmg-5 nsy-7 | 68 | 1.54 | 6.7e-03 |
| pTH5916 |  | efl-2 | 54 | 1.67 | 6.9e-03 |
| MSX2\_f1 |  | ceh-1 lin-39 eyg-1 alr-1 | 93 | 1.40 | 6.9e-03 |
| V$GATA1\_02 |  | elt-1 | 75 | 1.49 | 6.9e-03 |
| pTH10714 |  | nhr-142 nhr-84 nhr-216 | 72 | 1.51 | 7.0e-03 |
| pTH9108 |  | daf-12 nhr-5 | 83 | 1.44 | 7.2e-03 |
| pTH9261 |  | dmd-3 (0.62) lin-48 pax-3 | 108 | 1.33 | 7.3e-03 |
| pTH9164 |  | ceh-26 | 86 | 1.43 | 7.3e-03 |
| V$MYB\_Q6 |  | D1081.8 | 74 | 1.50 | 7.4e-03 |
| pTH10038 |  | gei-3 F56D1.1 | 69 | 1.53 | 7.7e-03 |
| pTH9323 |  | lin-1 tbx-39 odr-7 | 64 | 1.57 | 7.9e-03 |
| pTH9215 |  | C34D1.1 | 112 | 1.31 | 8.0e-03 |
| Nkx6-3\_3446 |  | cog-1 (0.7) | 98 | 1.37 | 8.0e-03 |
| Cart1\_1275 |  | lim-7 ceh-18 alr-1 | 82 | 1.44 | 8.1e-03 |
| pTH9279 |  | Y116A8C.22 | 140 | 1.22 | 8.3e-03 |
| pTH9969 |  | pag-3 | 101 | 1.35 | 8.3e-03 |
| pTH10624 |  | mef-2 mel-28 Y61A9LA.9 Y116A8C.22 | 114 | 1.30 | 8.5e-03 |
| pTH10028 |  | nhr-204 | 84 | 1.43 | 8.7e-03 |
| pTH9198 |  | dmd-3 (0.62) | 64 | 1.56 | 9.2e-03 |
| Smad3\_3805 |  | daf-8 | 89 | 1.40 | 9.5e-03 |
| Hoxd13\_2356 |  | pal-1 (0.55) | 116 | 1.29 | 9.6e-03 |
| Hoxa13\_3126 |  | pal-1 (0.55) ceh-24 | 112 | 1.31 | 9.9e-03 |
| pTH6569 |  | ceh-43 | 73 | 1.49 | 1.0e-02 |
| Hoxd1\_3448 |  | ceh-12 | 72 | 1.49 | 1.0e-02 |
| VENTX\_1 |  | pha-2 | 81 | 1.44 | 1.0e-02 |
| NKX28\_f1 |  | ces-1 ceh-24 C34H4.5 T22H9.4 | 66 | 1.53 | 1.0e-02 |
| MA0579.1 |  | pax-2 D1081.8 | 67 | 1.53 | 1.0e-02 |
| Barx2\_3447 |  | ceh-43 | 78 | 1.45 | 1.0e-02 |
| CEBPE\_f1 |  | C48E7.11 R07H5.10 | 105 | 1.33 | 1.0e-02 |
| FLI1\_f1 |  | lin-1 | 75 | 1.47 | 1.0e-02 |
| pTH9173 |  | efl-2 | 66 | 1.53 | 1.0e-02 |
| gl\_SANGER\_5\_FBgn0004618 |  | che-1 (0.51) ces-1 | 74 | 1.48 | 1.0e-02 |
| pTH8985 |  | athp-1 | 67 | 1.52 | 1.1e-02 |
| Sox1\_2631 |  | sox-4 | 116 | 1.29 | 1.1e-02 |
| MA0386.1 |  | tbp-1 | 80 | 1.44 | 1.1e-02 |
| Nkx2-2\_2823 |  | dsc-1 (0.84) ceh-24 | 73 | 1.48 | 1.1e-02 |
| ems\_FlyReg\_FBgn0000576 |  | ceh-2 skn-1 | 92 | 1.38 | 1.1e-02 |
| pTH8982 |  | ceh-48 | 83 | 1.42 | 1.1e-02 |
| MA0470.1 |  | efl-1 F49E12.6 | 64 | 1.54 | 1.1e-02 |
| TLX1\_f1 |  | ceh-19 | 83 | 1.42 | 1.1e-02 |
| Hmx3\_3490 |  | ceh-9 | 83 | 1.42 | 1.2e-02 |
| pTH8863 |  | hmg-12 | 134 | 1.23 | 1.2e-02 |
| pTH2283 |  | odd-2 | 63 | 1.55 | 1.2e-02 |
| pTH6447 |  | ceh-19 | 55 | 1.62 | 1.2e-02 |
| pTH8983 |  | tag-347 | 48 | 1.70 | 1.2e-02 |
| ECC-1\_ERALPHA\_HudsonAlpha |  | nhr-6 nhr-71 | 78 | 1.44 | 1.3e-02 |
| HepG2\_HSF1\_Stanford |  | Y53C10A.3 | 79 | 1.44 | 1.3e-02 |
| MA0261.1 |  | nhr-255 lin-14 | 89 | 1.39 | 1.3e-02 |
| pTH6449 |  | ceh-43 | 73 | 1.47 | 1.3e-02 |
| PROX1\_1 |  | ceh-26 | 71 | 1.48 | 1.3e-02 |
| Hoxa9\_2622 |  | php-3 (0.62) lin-39 | 86 | 1.40 | 1.3e-02 |
| V$OCT1\_06 |  | ztf-9 ceh-18 | 130 | 1.24 | 1.4e-02 |
| Gata3\_1024 |  | elt-1 | 93 | 1.37 | 1.4e-02 |
| pTH9222 |  | mel-28 | 131 | 1.24 | 1.4e-02 |
| Pbx1\_3203 |  | ceh-20 (0.55) | 59 | 1.57 | 1.4e-02 |
| FOXI1\_f1 |  | lin-31 (0.74) ceh-20 (0.55) dro-1 | 69 | 1.49 | 1.5e-02 |
| rn\_SOLEXA\_5\_FBgn0259172 |  | lin-29 | 113 | 1.29 | 1.5e-02 |
| pTH5778 |  | egl-5 (0.71) | 81 | 1.41 | 1.6e-02 |
| pTH8998 |  | mab-3 (0.55) | 104 | 1.32 | 1.6e-02 |
| pTH9182 |  | tbx-39 | 151 | 1.18 | 1.6e-02 |
| Barhl1\_1 |  | ceh-31 | 21 | 2.41 | 1.7e-02 |
| pTH9080 |  | mnm-2 | 85 | 1.39 | 1.7e-02 |
| V$CREB\_01 |  | crh-1 | 96 | 1.34 | 1.7e-02 |
| V$AHR\_01 |  | ahr-1 aha-1 | 66 | 1.50 | 1.7e-02 |
| Sox15\_3457 |  | sox-4 | 106 | 1.31 | 1.8e-02 |
| pTH9880 |  | end-1 | 85 | 1.39 | 1.8e-02 |
| Hnf4a\_2640 |  | nhr-62 | 78 | 1.42 | 1.8e-02 |
| I$DRI\_01 |  | cfi-1 (0.54) | 105 | 1.31 | 1.8e-02 |
| Dlx5\_3419 |  | lin-39 ceh-43 ceh-45 ceh-18 | 90 | 1.37 | 1.8e-02 |
| Atf1\_3026 |  | crh-1 | 54 | 1.59 | 1.9e-02 |
| Hoxa10\_2318 |  | ceh-24 | 59 | 1.55 | 1.9e-02 |
| Bsx\_3483 |  | ceh-31 | 71 | 1.46 | 1.9e-02 |
| pTH9913 |  | skn-1 fos-1 | 99 | 1.33 | 2.0e-02 |
| MA0083.2 |  | unc-120 (0.94) | 94 | 1.34 | 2.0e-02 |
| MA0544.1 |  | gei-11 | 88 | 1.37 | 2.0e-02 |
| MA0032.1 |  | let-381 | 57 | 1.56 | 2.0e-02 |
| pTH9085 |  | nhr-42 | 70 | 1.46 | 2.1e-02 |
| Hoxc8\_3429 |  | lin-39 | 92 | 1.35 | 2.1e-02 |
| V$GATA6\_01 |  | elt-1 | 86 | 1.38 | 2.1e-02 |
| HeLa-S3\_ZNF274\_UCD |  | C28G1.4 | 110 | 1.29 | 2.1e-02 |
| Nkx6-1\_2825 |  | cog-1 (0.7) | 81 | 1.40 | 2.2e-02 |
| Ets97D\_SANGER\_10\_FBgn0004510 |  | lin-1 | 104 | 1.30 | 2.3e-02 |
| pTH4325 |  | ceh-18 | 117 | 1.26 | 2.3e-02 |
| Tbp\_pr781 |  | tbp-1 | 117 | 1.26 | 2.4e-02 |
| Tcf2\_0913 |  | hmbx-1 | 70 | 1.45 | 2.4e-02 |
| pTH9708 |  | ceh-34 | 76 | 1.42 | 2.5e-02 |
| pTH9260 |  | mel-28 | 141 | 1.19 | 2.5e-02 |
| Srf\_3509 |  | unc-120 (0.94) | 84 | 1.38 | 2.5e-02 |
| RFX4\_1 |  | daf-19 | 73 | 1.43 | 2.5e-02 |
| Hoxb5\_3122 |  | lin-39 | 71 | 1.44 | 2.6e-02 |
| pTH6423 |  | pha-2 | 87 | 1.36 | 2.7e-02 |
| Hoxc4\_3491 |  | lin-39 | 78 | 1.40 | 2.8e-02 |
| pTH9237 |  | mel-28 | 101 | 1.31 | 2.8e-02 |
| pTH9934 |  | Y53H1A.2 | 135 | 1.21 | 2.8e-02 |
| POU4F3\_1 |  | unc-86 | 103 | 1.30 | 3.0e-02 |
| pTH9220 |  | mbr-1 | 97 | 1.32 | 3.0e-02 |
| Atf6\_SANGER\_5\_FBgn0033010 |  | atf-6 | 163 | 1.14 | 3.1e-02 |
| Hoxa5\_3415 |  | lin-39 | 72 | 1.42 | 3.6e-02 |
| Cdx1\_2245 |  | ceh-13 | 28 | 1.94 | 3.7e-02 |
| pTH2936 |  | nhr-239 | 82 | 1.36 | 3.8e-02 |
| Hoxa11\_2218 |  | php-3 (0.62) | 61 | 1.48 | 4.0e-02 |
| Hoxa7\_2668 |  | lin-39 | 85 | 1.35 | 4.1e-02 |
| Sox17\_2837 |  | sox-4 | 81 | 1.36 | 4.1e-02 |
| Ets98B\_SANGER\_10\_FBgn0005659 |  | lin-1 | 79 | 1.37 | 4.1e-02 |
| pTH9216 |  | ceh-18 | 86 | 1.34 | 4.4e-02 |
| I$DFD\_01 |  | lin-39 | 82 | 1.35 | 4.5e-02 |
| Tcfap2a\_2337 |  | aptf-1 | 37 | 1.72 | 4.6e-02 |
| pTH9958 |  | ztf-6 | 122 | 1.22 | 4.6e-02 |

### Correlated (and anti-correlated) transcription factors

|  |  |
| --- | --- |
| **Transcription factor** | **Correlation** |
| pat-9 | 0.96 |
| unc-98 | 0.96 |
| hlh-1 | 0.94 |
| K05F1.5 | 0.94 |
| unc-120 | 0.94 |
| nhr-69 | 0.93 |
| mls-1 | 0.88 |
| hlh-8 | 0.87 |
| rnt-1 | 0.87 |
| dsc-1 | 0.84 |
| K02D7.2 | 0.82 |
| nhr-19 | 0.78 |
| ehn-3 | 0.77 |
| elt-3 | 0.76 |
| nhr-111 | 0.75 |
| nhr-57 | 0.74 |
| lin-31 | 0.74 |
| tlp-1 | 0.72 |
| ceh-49 | 0.72 |
| hnd-1 | 0.71 |
| camt-1 | 0.71 |
| egl-5 | 0.71 |
| ceh-99 | 0.70 |
| nhr-9 | 0.70 |
| cog-1 | 0.70 |
| nhr-65 | -0.41 |
| unc-55 | -0.42 |
| nhr-71 | -0.42 |
| nhr-269 | -0.42 |
| D2030.7 | -0.43 |
| ztf-4 | -0.43 |
| wrm-1 | -0.43 |
| ceh-45 | -0.44 |
| nfya-2 | -0.44 |
| K11H3.4 | -0.44 |
| sox-3 | -0.45 |
| F21G4.5 | -0.46 |
| hlh-6 | -0.46 |
| nhr-38 | -0.46 |
| nhr-222 | -0.46 |
| ceh-32 | -0.46 |
| nhr-275 | -0.50 |
| madf-2 | -0.50 |
| pzf-1 | -0.51 |
| nhr-207 | -0.51 |
| sox-2 | -0.54 |
| nhr-215 | -0.54 |
| ccch-3 | -0.55 |
| snu-23 | -0.58 |
| ceh-53 | -0.62 |

### ChIP peaks enriched

|  |  |  |  |  |
| --- | --- | --- | --- | --- |
| **Gene** | **Experiment** | **Number of upstream peaks** | **Enrichment** | **FDR corrected p** |
| egl-5 | EGL-5\_Larvae-L3-stage | 46 | 3.06 | 6.8e-10 |
| nhr-129 | NHR-129\_Larvae-L2-stage | 60 | 2.46 | 1.6e-09 |
| lsy-2 | LSY-2\_Fed-L1-stage-larvae | 46 | 2.95 | 2.2e-09 |
| C34F6.9 | C34F6.9\_Larvae-L2-stage | 45 | 2.58 | 2.0e-07 |
| lsy-2 | LSY-2\_Larvae-L1-stage | 51 | 2.31 | 5.3e-07 |
| eor-1 | EOR-1\_Larvae-L3-stage | 43 | 2.45 | 2.1e-06 |
| fkh-2 | FKH-2\_Larvae-L3-stage | 24 | 3.54 | 6.2e-06 |
| nfya-1 | NFYA-1\_Late-Embryos | 39 | 2.44 | 1.0e-05 |
| unc-62 | UNC-62\_Larvae-L3-stage | 41 | 1.91 | 1.7e-03 |
| lin-35 | LIN-35\_Fed-L1-stage-larvae | 28 | 2.17 | 3.8e-03 |
| nhr-28 | NHR-28\_Larvae-L4-stage | 40 | 1.81 | 5.6e-03 |
| ama-1 | AMA-1\_Larvae-L3-stage | 28 | 2.03 | 1.0e-02 |
| sem-4 | SEM-4\_Larvae-L2-stage | 34 | 1.78 | 2.4e-02 |
| efl-1 | EFL-1\_Larvae-L1-stage | 26 | 1.96 | 2.7e-02 |
| ham-1 | HAM-1\_Fed-L1-stage-larvae | 51 | 1.50 | 4.6e-02 |
